# Increasing N Retention in Coastal Plain Agricultural Watersheds

**DOI:** 10.1100/tsw.2001.375

**Published:** 2001-12-13

**Authors:** Kenneth W. Staver

**Affiliations:** University of Maryland, College of Agriculture and Natural Resources, Wye Research and Education Center, Queenstown 21658, USA

**Keywords:** nitrate leaching

## Abstract

Historically, N availability has limited agricultural production as well as primary production in coastal waters. Prior to the middle of the last century, N available for grain production generally was limited to that supplied by previous legume crops, released from soil organic matter, or returned to the soil in animal wastes. The development of infrastructure to produce relatively low-cost inorganic N fertilizers eliminated the need to focus management of the entire agricultural system on increasing soil N availability. Increased N availability has contributed to dramatic increases in agricultural production but also has led to increased losses of both N and C from agricultural systems. N losses from cropland have been linked to increased algal production in the Chesapeake Bay, with N loss from cropland estimated to be the primary N input to the Bay from Coastal Plain regions of the watershed. The decade-long effort to reduce these losses has focused on reducing agricultural N use, but this strategy has yet to yield apparent reductions in N loadings to Coastal Plain tributaries. Although nitrate leaching losses are often attributed to inefficient use of N inputs, soil nitrate data indicate that both corn and soybeans can utilize nearly all available soil nitrate during periods of active growth. However, both crops tend to stop utilizing nitrate before mineralization has ceased, resulting in a late season buildup of root zone nitrate levels and significant leaching losses even when no N was applied. Reducing nitrate losses due to the inherent N inefficiency of summer annual grain crops will require the addition of winter annual crops to rotations or changes in weed management approaches that result in plant N uptake capacity being more closely matched to soil microbial N processes.

## INTRODUCTION

Excessive water column N availability in the Chesapeake Bay was identified in the early 1980s as a major cause of undesirable changes in the Bay ecosystem[1]. Both planktonic and epiphytic algal growth have increased due to elevated N inputs. The result has been expanded zones of oxygen-depleted bottom water during decomposition of summer algal blooms and an 80% decline in submersed vascular plant communities due to greater light interception by algal growth. As a result of the link established between high N availability and deleterious levels of algal growth, a key goal in the effort to restore the Chesapeake Bay has been reduction of N inputs. The initial goal established in 1988 was to reduce N inputs to the Bay by 40% by the year 2000.

Since agricultural activities were identified as a major source of total N inputs to the Bay, achieving the overall N reduction goal was highly dependent on reducing N losses from cropland. The focus on N loads from agricultural regions has been particularly intense in the Coastal Plain subwatersheds draining into the Bay from the east. Grain production—corn (*Zea mays* L.), soybeans (*Glycine max* L.), and wheat (*Triticum aestivum* L.) —is the dominant land use in these watersheds with much of the production used to supply feed to highly concentrated poultry operations. The level topography and soil drainage characteristics in these Coastal Plain watersheds enhance subsurface vs. overland discharge of excess precipitation (total precipitation - evapotranspiration). Elevated groundwater nitrate concentrations in association with agricultural activities have been documented throughout the region[2] and subsurface discharge of nitrate is the dominant route of N transport from cropland to the Bay[3,4].

Thus far, the effort to reduce subsurface N losses from cropland has primarily focused on educational and technical assistance programs to help farmers fine-tune N application rates so as to meet, but not exceed, crop N requirements. Implicit in this approach are the assumptions that farmers previously had been applying N at rates above the economic optimum and that reducing N applications to the economic optimum level would result in significant reductions in N losses from cropland. However, when the effort to restore the Chesapeake Bay began, little data to substantiate either of these assumptions existed for Maryland agricultural systems. Thus, although the approach has intuitive appeal as a first step in the process of reducing N losses from cropland, projecting reductions in N loads as a result of this effort is highly speculative.

Uncertainty regarding the ultimate reductions in N losses from cropland that can be achieved through fine-tuning of N inputs has been underscored in the Eastern Shore tributaries of the Chesapeake Bay. Despite the decade-long effort to reduce N losses, N levels in these tributaries have not decreased, and in some instances have shown an increasing trend[5]. The issue has been clouded somewhat by uncertainty surrounding the time required for changes in agricultural practices to affect N loads delivered to the Bay. Due to the high storage coefficients of shallow aquifers in the region, subsurface residence times of a decade or more have been reported, even in fields located directly adjacent to tidal waters[6]. Nevertheless, the apparent lack of progress toward meeting N discharge goals from predominantly agricultural watersheds precipitated legislation passed in 1997 that, in essence, converted from voluntary to mandatory the approach that had been in place to encourage reduced N applications. Unfortunately, the extent to which the approach will change N losses, even with complete compliance, is still unknown due to the lack of detailed data regarding past application rates. Moreover, although it has long been known that gross overapplications of N fertilizer will increase N leaching losses[7], it has been much more difficult to clearly establish that leaching rates change significantly with minor adjustments in applications likely to result from fine-tuning efforts. At this point, it is not clear that N losses from cropland will be cut to levels necessary to restore the Chesapeake Bay, even if all grain producers apply N at economically optimum levels.

Although removal of nitrate from subsurface flow in wetlands and riparian corridors down-gradient of cropland occurs in Coastal Plain settings in the Chesapeake Bay watershed[8,9], the benefit of this interception is already being realized. Despite high rates of subsurface nitrate attenuation in some watersheds[10], nitrate discharge rates to tidal waters from agriculture-dominated watersheds are still sufficient to fuel excess algal growth. There is an aggressive ongoing effort to encourage establishment of riparian buffers and restoration of wetlands to enhance nutrient attenuation mechanisms. However, despite the many benefits of these practices, hydrogeologic factors suggest that this effort will not substantially further reduce subsurface N losses[11]. Achieving long-term reductions of subsurface N losses of the magnitude necessary to restore the Chesapeake Bay will likely require adoption of agricultural practices that reduce nitrate leaching losses from the root zone.

Volumes have been written on how to manage soil N levels to enhance crop production and minimize nitrate losses[12,13,14]. Conceptually the approach is straightforward — limit nitrate availability in the soil during periods of water percolation. Where irrigation is used extensively, improved irrigation techniques can be used to reduce nitrate losses by minimizing water movement below the crop rooting zone[15]. In the Chesapeake Bay watershed, irrigation of grain crops is minor, leaving management of soil nitrate levels as the primary option for reducing nitrate leaching losses. Thus far, the most widely used approach for managing soil nitrate levels has been input management. In this approach, little is changed other than rates and timing of N application.

Acceptance generally is high because, thus far, the targeted N input level has been the economic optimum. In theory, a farmer will maximize profits by adopting this approach unless N surpluses exist due to concentrated animal production. Another approach is to modify cropping systems so as to minimize the accumulation of soil nitrate through closer coupling of plant uptake with soil N mineralization patterns. This approach requires more adjustments by the farmer and also can affect soil C and water cycles. In addition, cost of production may be increased, making voluntary adoption unlikely. This paper will consider soil N cycles and nitrate leaching patterns in the primary crop rotations used in the Coastal Plain region of the Chesapeake Bay watershed and assess the potential for various management options to yield significant reductions in N losses from cropland.

## PROCEDURES

The results presented in this study are from a series of experiments conducted in the Wye River drainage basin in Queen Anne's County, Maryland (38°55' N, 76°09' W). Soils in the study area (Typic and Aquic Hapludults) exhibit gentle slopes (0 to 3%) and are characterized as moderately well drained. The soil surface ranges from 4 to 6 m above sea level and the water table is located at a seasonally variable depth of 1 to 4 m below the soil surface. Precipitation is evenly distributed throughout the year and averages 105 cm/year.

Although experimental design varied slightly among the series of experiments carried out between 1985 and 2000, the overall objective of these experiments was to characterize soil nitrate dynamics in various crop management systems. A detailed description of leachate, soil, and plant tissue sampling and analysis techniques has been presented previously[16]. The crop management systems evaluated are typical of those used in the Coastal Plain region of the Chesapeake Bay watershed. Corn and soybean production were evaluated under winter-fallow conditions and also with a rye (*Secale cereale* L.) winter cover crop. Corn generally was planted in early May and harvested in late September. Approximately 20% of inorganic N applications to corn were at planting with the remainder being applied 30 to 50 days later. All manure was poultry house floor litter that was applied and incorporated into the soil just prior to corn or soybean planting, except for one experiment in which fall application was evaluated. Full-season soybeans were planted in late May and harvested in mid-October. A rotation of corn/wheat/double-crop soybeans also was evaluated. Wheat generally was planted in mid-October and harvested in late June or early July with double-crop soybeans being planted immediately after wheat harvest. N was applied to wheat as a single surface application in early April.

Soil nitrate levels were determined colorimetrically on 2 M KCl extracts from oven-dried samples. Root zone leachate was monitored using gravity lysimeters installed approximately 60 cm below the soil surface. Lysimeters were constructed from 1.8-m sections of 5-cm diameter PVC well casing slotted on one side for a distance of approximately 80 cm. Lysimeters were installed parallel to the soil surface through the wall of a 1.2-m deep pit. Installation required no disturbance of the soil profile above the collection area and allowed execution of agronomic activities over the collection area using commercial scale equipment. Leachate was collected immediately after flow ceased, which usually occurred within 24 h of when precipitation ended. Leachate nitrate concentrations were determined using high-pressure chromatography.

## RESULTS AND DISCUSSION

Results from detailed hydrologic studies conducted at the site have been presented previously[16,17], but will be briefly summarized here because they are critical in determining leaching losses of nitrate.

Potential evapotranspiration by established summer annual crops generally exceeds precipitation inputs during most of the growing season. As a result, the root zone tends to be maintained at well below maximum water holding capacity for most of the summer growing season, with little movement of water and solutes occurring across the lower boundary of the root zone. This lack of summer recharge to shallow aquifers is reflected in falling water tables and decreasing streamflow during summer months, with annual minimum levels typically occurring in early autumn. Root zone water deficits are gradually replenished during autumn months as evapotranspiration rates decrease, and most significant precipitation events from late fall through late spring result in movement of water and leaching of available solutes from the root zone. Thus, soil nitrate concentrations during the growing season are highly relevant to crop production, but have little effect on nitrate leaching losses. It is soil nitrate concentrations entering the late fall/winter period of frequent leaching events that primarily determine annual subsurface nitrate losses from grain fields.

The balance between processes supplying and removing nitrate from the soil during the growing season determines postharvest root zone nitrate concentrations. However, annual variability in temperature and precipitation patterns makes it difficult to precisely balance growing season root zone N availability with plant uptake. Total N applications are based on yield projections, but actual yields vary widely from year-to-year due to rainfall and temperature patterns. Because N applications occur prior to the critical period when actual yields are determined and most N uptake occurs, it is impossible to precisely match N applications to crop N uptake. The result is widely varying rates of grain production per unit of N input even within the same field under the same management system (Fig. 1).

However, low harvest efficiency of applied N (N in harvested grain per unit of applied N) did not necessarily translate into proportional increases in postharvest root zone nitrate concentrations. The highest postharvest soil nitrate level (114 kg/ha) did occur following the lowest grain harvest in the 10-year study. During that year moisture deficits occurred early in the growing season, sharply reducing vegetative growth and leading to premature senescence of the crop. But other than that single extreme case, postharvest root zone nitrate concentrations were not found to be strongly dependent on grain yields (Fig. 1). Reduced grain yields in most years resulted from moisture deficits during critical periods later in the growing season. N uptake, as indicated by postharvest soil nitrate concentrations, apparently was not greatly affected. When grain yields were near or above the target level of 8.8 Mg/ha, postharvest soil nitrate levels were al-

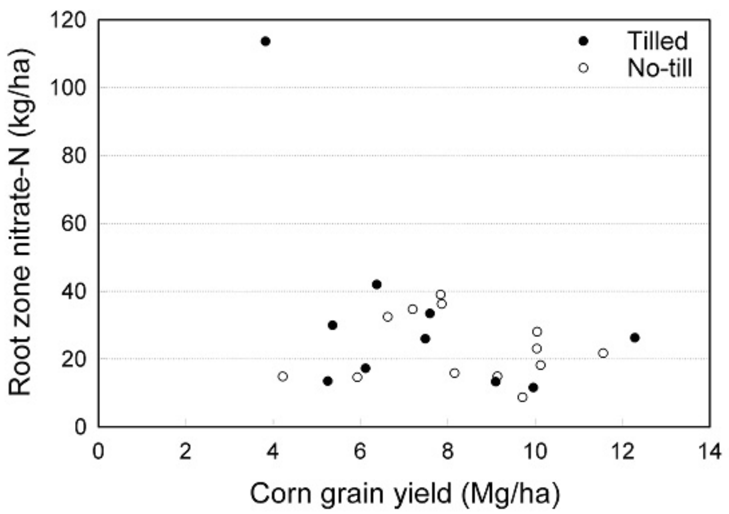

**FIGURE 1.** Root zone (0 to 60 cm) soil nitrate (n = 5) following grain harvest in fields in continuous corn production from 1988–1998. All fields received 158 kg/ha of inorganic N annually.

ways less than 30 kg/ha, but not reduced to the extent expected based on fertilizer N use efficiency.

In several years, more intensive root zone sampling was conducted during the growing season to discern why a significant pool of leachable nitrate remained in the root zone even when grain production goals were met (Fig. 2). In all years corn was found to be able to reduce soil nitrate-N concentrations in the top 30 cm of the root zone to less than 1 mg/kg by the end of the period of peak uptake (Fig. 2) in plots where N was applied in inorganic forms and also in poultry manure. However, after uptake ceased, typically by early September, soil nitrate concentrations gradually increased until mid-autumn, even where no N had been applied. This suggests that postharvest nitrate levels in many years were not determined by utilization efficiency of applied N, but rather reflected inefficient use of nitrate released in the root zone late in the growing season.

Soil nitrate patterns in soybean fields followed the same general trend as in cornfields without the spike resulting from N fertilizer application (Fig. 3). Soybeans are grown both as a full-season crop and also as a double-crop planted in early summer after harvest of winter cereals. Because full-season soybeans are planted well after soil temperatures have reached levels that favor soil microbial activity, nitrate does accumulate in the root zone early in the growing season after weed growth is suppressed with herbicides or tillage. Similarly, nitrate also accumulated during the gap in plant N uptake between when wheat had matured and N uptake capacity by the following soybean crop had become significant. Even though soybeans can obtain N via fixation, they took up most of the nitrate available in the root zone, although soil nitrate levels were not reduced to levels as low as those observed in corn fields (Fig. 2). Because soybeans generally are planted later and grow actively later in the growing season than corn, the period of increasing soil nitrate after crop uptake has stopped is shorter. This is especially true for double-crop soybeans planted after wheat harvest, which typically grow actively into early autumn. However, even though the late season gap in plant N uptake was shorter, soil nitrate concentrations increased to levels as high as those in cornfields in which yield goals were met.

Utilization of soil nitrate by winter cereals and the resulting effect on nitrate leaching patterns has been studied extensively at the site and reported previously[16,18,19]. Wheat is the primary

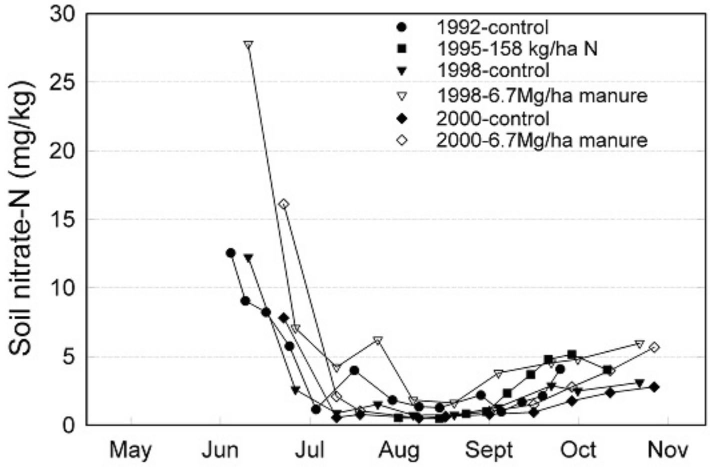

**FIGURE 2.** Surface soil (0 to 30 cm) nitrate concentration (dry mass basis, n = 3) in corn plots receiving various N inputs. The manure was floor litter from poultry houses that contained 36 kg/Mg of total N in 1998 and 30 kg/Mg of total N in 2000.

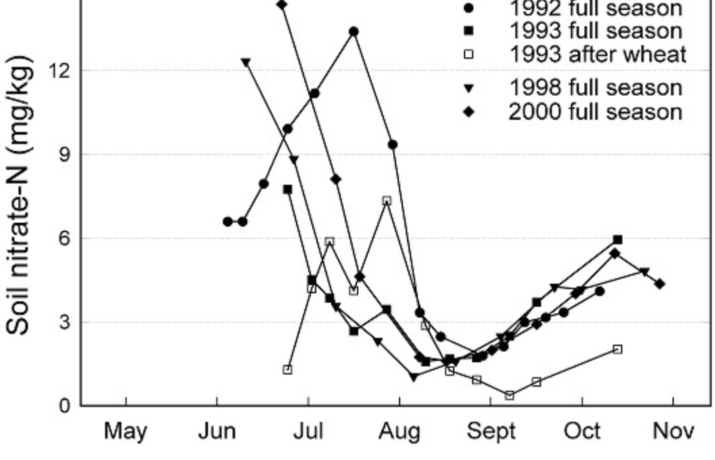

**FIGURE 3.** Surface soil (0 to 30 cm) nitrate concentration (n = 3) in unfertilized soybean plots.

winter cereal grown in the region with rye also being planted as a winter cover crop after harvest of corn and soybeans. Generally little or no N is applied at planting and growth prior to the onset of winter is limited by temperature or moisture. For early planted winter cereals, fall growth can be limited by N availability. Because little additional nitrate is released from the root zone from mid-autumn until early spring, winter cereals can draw root zone nitrate concentrations down to very low levels. The amount of soil nitrate utilized in the fall depends on how early planting occurs, temperature, and the extent of leaching relative to rates of uptake. Most N is applied to wheat in late winter or early spring just prior to the period of maximum uptake. Like corn, cereals were found to reduce soil nitrate concentrations to very low levels. However, winter cereals have the potential to maintain soil nitrate concentrations at low levels from late autumn until late spring, as compared to the relatively brief summer period of N uptake by corn and soybeans.

Most waste from poultry operations in the region is applied to cropland. Where poultry production is less concentrated, poultry manure primarily is applied to corn, but applications to all crops occur in regions of concentrated production. In some instances, manure is fall applied due to a lack of storage. The patterns of root zone nitrate concentrations where poultry manure was applied to cornfields were similar to those where inorganic N was applied, except that late season rates of nitrate accumulation tended to be enhanced (Fig. 4). Applications to soybean fields increased early season peaks in nitrate concentrations as well as rates of nitrate accumulation during early autumn. Ammonia in poultry manure applied in early fall apparently was rapidly nitrified, resulting in sharp increases in soil nitrate concentrations (Fig. 5). Rye planted just after poultry manure application in early autumn reduced surface soil nitrate concentrations to levels below those in winter-fallow fields not receiving waste applications, but not until late winter.

Leachate nitrate concentrations generally reflected patterns of root zone soil nitrate. Despite annual variability in weather, leaching patterns were generally similar from year to year (Fig. 6). Leaching rarely occurred during summer months, increased during autumn, and occurred as a result of most major precipitation events during winter and early spring. In the absence of severe drought conditions, leachate nitrate-N concentrations following corn and soybeans were not noticeably different, both tending to range from 5 to 15 mg/l when fields remained fallow between growing seasons. During the severe droughts of the late 1980s, nitrate-N concentrations following corn production ranged from 20 to 40 mg/l[16]. Although the effects were not quantifiable, observation suggests that leachate nitrate concentrations following full-season soybeans in 1994 (Fig. 6b) were reduced under winter-fallow conditions relative to other years due to late season weed growth in the leachate collection area. Where winter cereals were planted either as cover crops or for harvest, leachate nitrate concentrations tended to decrease during autumn and early winter and remain at low levels (<0.1 mg/l) from midwinter through early spring. Earlier corn harvest relative to soybeans allowed earlier planting of cereals and more complete removal of nitrate from the root zone by the time the major leaching period began. Leachate nitrate concentrations increased in late spring after herbicides were applied to cover crops. In April 1993 leaching continued after N application to wheat resulting in briefly elevated leachate nitrate concentrations (Fig. 6c). However, leachate nitrate concentrations in the rotation, including wheat (Fig. 6c), tended to be lower than in the corn-soybean rotation (Fig. 6b), despite greater N inputs.

Leaching patterns where organic wastes were applied also were consistent with soil nitrate concentrations (Fig. 5). Although soybeans utilized a large fraction of nitrate released from spring-applied poultry manure, leachate nitrate concentrations the following winter were well above those where no manure had been applied. Rapid release of nitrate from fall-applied manure resulted in the highest leachate nitrate concentrations observed in any studies at this site during the last decade (Fig. 5b). In 1990 soybeans were planted into a killed alfalfa sod, which probably

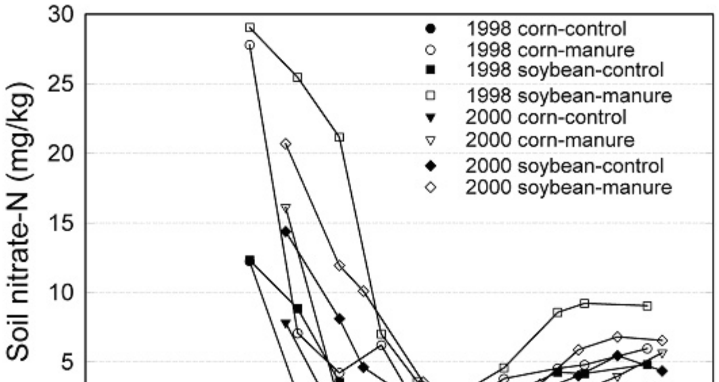

**FIGURE 4.** Surface soil (0 to 30 cm) nitrate concentration (n = 3) in corn and soybean plots receiving no N and poultry manure at a rate of 6.7 Mg/ha. The manure applied contained 36 kg/Mg of total N in 1998 and 30 kg/Mg of total N in 2000.

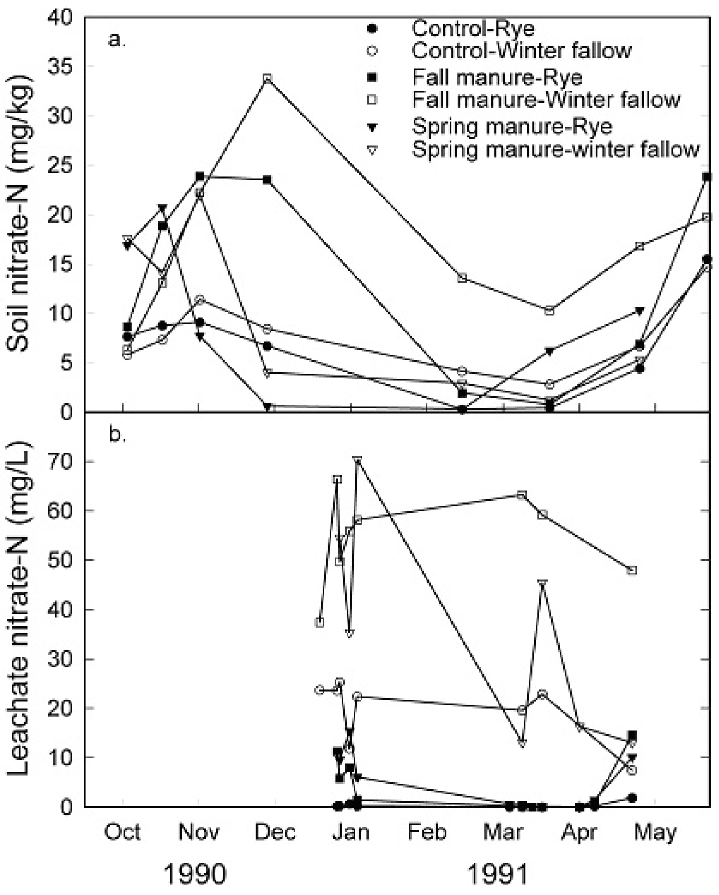

**FIGURE 5.** Soil (0 to 30 cm, n = 3) and leachate (collected at a depth of 60 cm, n = 6) nitrate in soybean plots planted with soybeans in 1990. Spring manure was applied on June 12 just prior to planting and fall manure was applied on October 18. Manure was applied at a rate of 9.5 Mg/ha and contained 34 kg/Mg of total N. Rye was planted on October 20.

accounts for why postharvest soil and leachate nitrate concentrations where no poultry manure was applied were well above those observed in soybean fields in later studies (Figs. 5 and 6). The effect of winter cereals was consistent in all treatments. Below average precipitation in the fall of 1990 delayed leaching until the end of the year, by which time cereals had removed most of the nitrate from the root zone even where manure had been fall applied.

## MANAGEMENT IMPLICATIONS

Reducing subsurface losses of nitrate requires close coupling of plant demand with nitrate availability. Although N applications increase the potential for nitrate leaching, periods of high plant N demand tend to occur when evapotranspiration rates also are elevated. Applying N just prior to periods of peak demand will result in elevated soil nitrate concentrations only briefly, during a period when the likelihood of leaching is low due to high crop water use. All the crops included in this study were capable of utilizing nearly all of the nitrate that was available in the root zone during periods of active uptake. Thus, if N is not supplied at excessive rates, uptake can be nearly complete. For the last decade this has been the primary strategy in the effort to reduce N losses from cropland in the Chesapeake Bay watershed — apply the economically optimum rates of N as close to the period of peak demand as possible. For inorganic N, this has been primarily an educational program to discourage overapplication and encourage timely applications. For organic N sources, there has been a technical assistance program focused on testing of wastes and soils to determine proper application rates, and cost-share programs to increase manure storage capacity so as to minimize the need to apply wastes to cropland at times not dictated by crop needs. The major unanswered question is whether this strategy alone will result in the reductions in N losses from cropland necessary to restore water quality in Chesapeake Bay. Because the role of educational programs in modifying N application rates and timing is largely unknown, it is difficult to determine the levels of reduction that will be achieved as a result of efforts thus far. Results from this study suggest that unless pre-1988 N application rates for corn were well above economically optimum levels, the reductions in N losses that will be achieved will be minor. Because extremely high nitrate leaching rates can occur when organic wastes are fall applied or applied at excessive rates, programs to help farmers avoid these practices have the potential to significantly reduce overall N losses. As with programs focusing on inorganic N rates, how these programs have affected manure spreading practices is largely unquantified.

Even though optimum management of N inputs is the logical first step to reducing N losses from cropland, the reductions

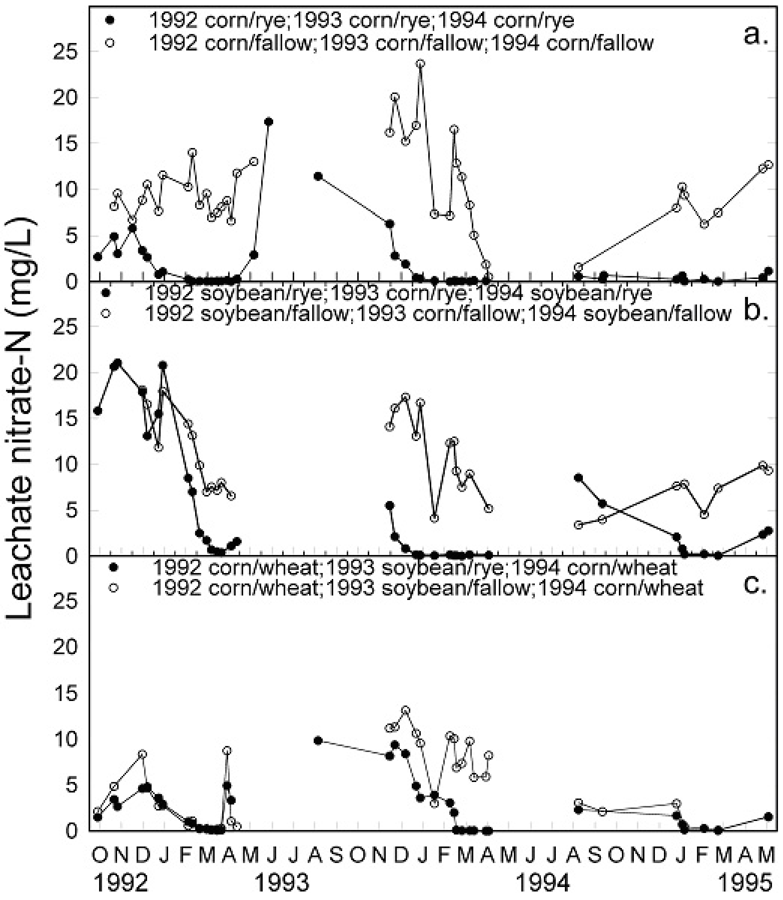

**FIGURE 6.** Leachate (collected at a depth of 60 cm) nitrate concentrations (n = 6) in the major cropping sequences in the Maryland Coastal Plain. Rye was planted immediately after grain harvest and sprayed with herbicide in early April. Wheat was planted in mid-October and harvested in late June. Corn plots received 158 kg/ha of inorganic N and wheat plots 90 kg/ha in early April. Symbols indicate days of leachate flow.

achievable using this strategy are limited. The most obvious limitation of this approach is that it has little effect on losses from soybean fields because N inputs are minimal. Even though inputs can be managed so that use of root zone nitrate is highly efficient during the growing season, failure of summer annual crops to utilize nitrate released late in the growing season results in significant postharvest root zone nitrate levels. Organic wastes (even when applied at judicious rates) and drought conditions during the growing season further widen the gap between N mineralization rates and crop uptake late in the growing season. Largely as a result of inefficient use of late season nitrate releases, results from this study suggest that long-term average annual nitrate leaching losses in mixed corn and soybean production systems in the Coastal Plain region of the Chesapeake Bay watershed will exceed 20 kg/ha despite fine-tuning of N inputs.

Reducing nitrate leaching losses to levels below those obtainable through input management will require management changes that lead to closer coupling of plant N uptake with patterns of soil nitrate release. Nitrate accumulates early in the growing season in the gap between when weeds are suppressed and significant uptake capacity by the summer annual crop develops, and at the end of the season after uptake capacity diminishes. Most of the nitrate released early in the growing season will eventually be utilized by the summer annual crop, but nitrate that accumulates at the end of the growing season is most likely to be leached out of the root zone. Winter cereals can provide uptake capacity for nitrate not utilized by summer annual crops. Nitrate uptake is not matched as closely to release as it is in perennial systems, but the approach works well for reducing nitrate leaching where soil moisture holding capacity is sufficient to retain nitrate in the root zone until fall-planted cereals can utilize it. Using plant uptake to retain N also will increase the supply of plant residues to the soil. In the case of winter cereals, the quantity of additional plant residues will depend on N availability and how long the crop is allowed to grow the following spring[16].

The retention of N through accumulation of soil organic matter will have both short- and long-term effects on soil N cycles. Even though total soil N increases gradually when winter cereals are used to reduce nitrate leaching[20], effects on N release during the growing season are minor and can be more than offset by the ability of cereals to purge nitrate from the entire root zone[16]. When cereals are allowed to grow until late spring, the additional high C residues may make it necessary to increase inorganic N application rates to prevent N deficiency in following corn crops. This situation is similar to the soil C-related reductions in N availability that often occur during transitions to reduced tillage systems[21]. In both situations, as soil C

concentrations increase there is a short-term microbial demand for inorganic N. However, the additional N inputs that may be needed to meet crop needs are minor relative to the massive quantities of N that have been lost from undisturbed soils converted to grain production[22].

## CONCLUSIONS

The central focus of the effort to restore the Chesapeake Bay is a reduction in nutrient loadings. In the concentrated grain-producing areas in the Coastal Plain region of the Bay watershed, N losses through subsurface flowpaths account for a major fraction of N inputs to tidal waters. These N losses can be divided into two general categories — losses resulting from N inputs greater than what is needed for optimum crop production and losses resulting from the inherent inability of annual grain crops to efficiently utilize soil nitrate. Thus far in the decade-long effort to reduce N losses from cropland, the primary focus has been on fine-tuning inputs to match crop needs. Although this approach offers some potential to reduce losses, especially where animal wastes are applied to cropland, there is little apparent evidence to date that this approach alone will reduce losses from cropland to the extent deemed necessary to restore the Chesapeake Bay. Even though both corn and soybeans can utilize almost all of the nitrate available in the root zone in midsummer, uptake capacity diminishes late in the growing season before soil temperatures reach levels low enough to curtail soil microbial processes. In the absence of plant uptake, nitrate accumulates at the end of the growing season, thereby increasing the potential for leaching losses during the upcoming winter. Thus, although nitrate leaching is often attributed to inefficient use of N inputs, in the systems evaluated in this study it was more a consequence of inefficient use of soil nitrate.

Reducing the leaching potential resulting from late season mineralization will require that plant uptake capacity be maintained to match microbial release rates. In essence, a strategy is needed that makes annual crop systems mimic undisturbed perennial systems in which there is persistent uptake capacity for nitrate released in the soil. In the Coastal Plain region of the Chesapeake Bay watershed, winter cereals can be used to compensate for the inability of summer annual crops to fully utilize soil nitrate, whether it results from excessive or ill-timed N inputs or late-season mineralization in well-managed systems. Although nitrate released late in the growing season is not utilized immediately, precipitation patterns and soil moisture holding characteristics combine to retain nitrate in the root zone long enough for uptake by winter cereals to eventually occur. In settings where nitrate is lost more rapidly from the root zone, a more aggressive approach to providing uptake capacity will be necessary to reduce nitrate leaching losses. In these settings, voids in uptake capacity will need to be shortened using either overseeding of cover crops or weed management strategies that provide almost continuous uptake capacity. However, unless strategies for reducing nitrate leaching losses provide some economic advantage through higher soil moisture holding capacity or pest suppression, adoption will be limited without incentives or regulatory pressure.

| | | |
|---|---|---|
| Received: | August | 14, 2001 |
| Revised: | November | 2, 2001 |
| Accepted: | November | 9, 2001 |
| Published: | December | 13, 2001 |
